# Nano-drug delivery systems (NDDS) in metabolic dysfunction-associated steatotic liver disease (MASLD): current status, prospects and challenges

**DOI:** 10.3389/fphar.2024.1419384

**Published:** 2024-08-06

**Authors:** Ying Yang, Xiaojing Wang

**Affiliations:** Department of Gastroenterology, The Fifth Affiliated Hospital of Wenzhou Medical University and Lishui Municipal Central Hospital, Lishui, China

**Keywords:** metabolic dysfunction-associated steatotic liver disease, MASLD, nano-drug delivery system, NDDS, nanocarrier, nanoparticle, nanoliposome

## Abstract

About one-third of the global population suffers from metabolic dysfunction-associated steatotic liver disease (MASLD), but specific treatments for MASLD have long been lacking, primarily due to the unclear etiology of the disease. In addition to lifestyle modifications and weight loss surgery, pharmacotherapy is the most common treatment among MASLD patients, and these drugs typically target the pathogenic factors of MASLD. However, bioavailability, efficacy, and side effects all limit the maximum therapeutic potential of the drugs. With the development of nanomedicine, recent years have seen attempts to combine MASLD pharmacotherapy with nanomaterials, such as liposomes, polymer nanoparticles, micelles, and cocrystals, which effectively improves the water solubility and targeting of the drugs, thereby enhancing therapeutic efficacy and reducing toxic side effects, offering new perspectives and futures for the treatment of MASLD.

## 1 Introduction

MASLD is defined as a complex and progressive metabolic disorder that typically begins with excessive fat accumulation in the liver, evolving into metabolic dysfunction-associated steatohepatitis (MASH), and even leading to hepatic failure or hepatocellular carcinoma (HCC) (Chalasani et al., 2012; Hendriks et al., 2023). The disease was once known as non-alcoholic fatty liver disease (NAFLD), and in 2023, a consensus group led by three large pan-national liver associations voted to approve the proposal to change its name to MASLD (Rinella et al., 2023). Clinically, MASLD patients are characterized by elevated triglycerides (TGs), increased low-density lipoproteins, and decreased high-density lipoproteins (Deprince et al., 2020), and are often accompanied by other metabolic disorders such as obesity, diabetes, and abnormal blood pressure (Pouwels et al., 2022; Rong et al., 2023). In the 30 years from 1990 to 2019, the global comorbidity rate of MASLD increased from approximately 25%–38% (Younossi et al., 2023). It is expected that by 2030, advanced liver disease cases and liver related mortality caused by MASLD will be more than double (Estes et al., 2018). Obviously, MASLD has become an unignorable worldwide healthcare issue.

Although MASLD is one of the most common liver diseases, its exact pathogenesis remains unclear. The early classic “two-hit” theory suggests that MASLD development initially results from factors such as insulin resistance (IR), unhealthy lifestyles, and high-fat diets (HFD) leading to excessive accumulation of liver fat, which constitutes the first hit. The oxidative stress induced by fat accumulation serves as the second hit to the liver, ultimately bringing about MASH and fibrosis (Day and James, 1998; Tilg et al., 2016; Chen Z. et al., 2020a). Nowadays, more researchers believe that in addition to IR and oxidative stress, hormones secreted by adipose tissue, genetic and epigenetic factors, gut microbiota, and nutrients also play important roles in the development of MASLD disease, which is known as the “multiple-hit” theory (Tsochatzis et al., 2009; Anstee et al., 2011; Kirpich et al., 2015; Buzzetti et al., 2016).

However, while the multiple-hit theory provides a more reasonable explanation for the progression of MASLD(16), it still cannot accurately describe the pathological mechanisms of MASLD, which remains the biggest obstacle to the effectiveness of MASLD treatments. Some MASLD patients are recommended to control the disease by lifestyle changing [e.g., diet control (Akbulut et al., 2022) and increased physical exercise (Huber et al., 2019)] or bariatric surgery (Pratt et al., 2018), but more patients still rely on drug therapy. Unfortunately, the specific drugs for MASLD have long been missing (Muthiah and Sanyal, 2020). Currently, MASLD pharmacotherapy mainly targets the pathogenic factors, indirectly achieving control of disease progression through treating type 2 diabetes mellitus (Della et al., 2021), improving oxidative stress (Sanyal et al., 2010), and reducing blood lipids (Kim et al., 2017), but the efficacy is often poor. Moreover, some drugs also have disadvantages such as low bioavailability (Jiang et al., 2020) and obvious side effects (Cusi, 2020), further limiting their therapeutic effectiveness. Recently, FDA approved for the first time a drug called Rezdiffra (resmetirom) for the treatment of adult MASH with advanced liver fibrosis (Ledford et al., 2024), which is almost the only drug specifically designed for MASLD treatment, providing encouraging hope for further drug development.

In recent years, nano-drug delivery systems (NDDSs) have garnered widespread attention in the research of various diseases, especially cancer. With advantages such as good biocompatibility, low side effects, precise targeting ability, and controlled release properties (Zhu et al., 2019; Chen S. et al., 2020b; Lan et al., 2021; Sun et al., 2021), NDDS offers promising prospects in the field of drug therapy. Reported delivery systems mainly include cocrystals, liposomes, polymer nanoparticles, self-nanoemulsifying drug delivery systems, nanosuspensions, and amorphous solid dispersions (Joshi et al., 2018; Jiang et al., 2020), which can form nanostructures via loading, adsorption, bonding, and other methods to carry drugs (Zhou et al., 2016). Several studies have focused on incorporating NDDS into the treatment of MASLD. While most of these studies are still in the animal experimentation stage and have not yet entered clinical trials, the potential clinical benefits of this practice are foreseeable in the future.

This article will review the current research status of NDDS in the treatment of MASLD based on the possible pathogenesis of the disease, elucidate how nanomaterials optimize drug properties, enhance drug efficacy, and evaluate the clinical application prospects of NDDS, providing reference for further research in this field.

## 2 NDDS in MASLD pharmacotherapy

The complex pathogenesis of MASLD involves various genetic, metabolic, and environmental factors, whose parallel effects collectively cause liver damage (Moghtadaie et al., 2023). Targeted drug therapy aimed at the pathogenesis of MASLD is an ideal way to control disease progression. Addressing certain known aspects of MASLD pathogenesis, some studies have utilized low-toxicity nanomaterials to load drugs, increasing drug biocompatibility and resisting degradation, assisting drugs in locating the corresponding sites for sustained release (Zhang et al., 2013; Bose et al., 2021), fully exploiting drug efficacy, which provides new insights into MASLD treatment.

When nanoparticles enter the bloodstream, they are quickly enveloped by serum proteins, triggering the mononuclear phagocytic system (MPS) to recognize and engulf them through various receptors (Gustafson et al., 2015). MPS includes resident tissue macrophages in different organs, mainly in the liver and spleen, as well as blood monocytes, dendritic cells, and their bone marrow progenitor cells (Hume, 2006). In addition, increasing evidence suggested that scavenger endothelial cells (such as liver endothelial cells) also play an important role in the process of removing nanoparticles. They constitute the major contact surface with blood and largely affects nanoparticles exiting the bloodstream (Wen et al., 2023). These characteristics severely limit the efficacy of the loaded drug for other applications of NDDS, but for the treatment of liver diseases, it is a natural advantage of nanoparticles.

Table 1 lists some research examples. Although most related studies are currently in the stage of cell experimentation or small animal model verification, the excellent properties exhibited by these nanomaterials are promising.

**TABLE 1:** Preclinical studies on the use of nano-medicine delivery systems for the treatment of MASLD/MASH.

Drug type	Drug loaded	Delivery system	Component(s)	Size (nm)	*In vitro*/vivo model	Administration	Ref
Insulin sensitizer	Silibinin	Nanoliposome	Sesame oil, precirol ATO5 and soy phosphatidylcholine	225.49 ± 17.18	HFD-induced MASLD mice	Oral	Chen et al. (2018b)
		Nanoliposome	Phospholipids and cholesterol	119.76	HFD-induced MASLD mice	Oral	Cai et al. (2023)
		Nanoliposome	DPPC/POPC-cholesterol-cholic acid	100	HFD-induced MASLD mice FFA-treated HepG2 cells	Oral	Yan et al. (2023)
		SNEDDS	Propylene glycol caprylate	83.89 ± 39.02	HFD-induced MASLD mice	Oral	Chen et al. (2018b)
	Resveratrol	Nanoparticle	PLGA	176.1	Oleic acid-treated HepG2 cells	NA	Wan et al. (2018)
		Micelle	Lysozyme micelles and D-galactose	50	Palmitic acid and Oleic acid-treated HepG2 cells HFD-induced MASLD mice	Intravenous	Teng et al. (2019)
	Naringenin	Nanoliposome	Soybean lecithin and cholesterol	98 ± 5	MCD diet-induced MASLD mice	Oral	Chen et al. (2017)
		Nanoliposome	Soybean lecithin, stearic acid, monostearin and oleic acid	162.9 ± 11.7	MDCK cells MCD diet-induced MASLD mice	Oral	Hu et al. (2021)
		Cocrystal	Isonicotinamide	NA	MCD diet-induced MASLD mice	Oral	Jiang et al. (2020)
Lipid-lowering drug	Deoxyschizandrin	Nanoliposome	L-α-phosphatidylcholine and cholesterol	73.08	3T3-L1 preadipocytes BALB/c-nu mice	Intravenous	Liu et al. (2018)
	Platensimycin	Nanoliposome	PLGA and PEG	NA	Palmitic acid and Oleic acid-treated HepG2 cells Western diet-induced MASLD mice	Intravenous	Su et al. (2021)
	Berberine & Curcumin	Nanoparticle	Salts-sodium deoxycholate, soybean lecithin, cholesterol and octadecylamine	150	Caco-2 and LO2 cells High fat and sucrose diet-induced MASLD mice	Oral	Chen et al. (2021)
	Rapamycin	Nanoparticle	mPEG-PLGA	130.4 ± 17.3	HFD-induced MASLD mice	Intravenous	Zhao et al. (2020)
	Inositol hexanicotinate	Micelle	Glycyrrhizic acid and arabic gum	145.8	Sprague Dawley rats HFD + tetracycline induced MASLD mice	Oral	Zheng et al. (2021)
Antioxidant	Silymarin	Nanoliposome	Soybean lecithin and DSPE-PEG 2000	286.5 ± 23.8	Fat-emulsion-treated HepG2 and Caco-2 cells PNPLA3 I148M transgenic MASLD mice	Oral	Liang et al. (2018)
	Lycopene	Nanoliposome	Phsopholipon 90 G, cholesterol and lycopene	250.2 ± 15.26	HFD-induced MASLD rats	Oral	Salem et al. (2023)
	Oridonin and LY294002	Nanoliposome	DOPC, cholesterol and DSPE–PEG2000	125.47 ± 2.11	AML-12 and LX-2 cells CCL4-induced MASLD mice	Intravenous	Xin et al. (2023)
	Curcumin/Calcitriol	Nanoliposome	Egg phosphatidylcholine and cholesterol	NA	Human liver perfusate cells MCD diet-induced MASH mice	Intravenous	Maradana et al. (2018)
	Nitroxide radicals (TEMPO)	Micelle	Methoxy-poly (ethylene glycol)-b-poly (4 chloromethylstyrene)	36–37	CDAA diet-induced MASH mice	Oral	Eguchi et al. (2015)
	Celastrol	Micell	mPEG–PCL and celastrol	50–70	HFD-induced obese mice	Oral	Zhao et al. (2019)
		Micelle	PBE or PFM	202.5 ± 1.5	Palmitic acid and Oleic acid-treated HepG2 cells HFD-induced MASLD rats	Intravenous	Pan et al. (2023)
	SKLB023	Micelle	Sodium glycocholate and egg phosphatidylcholine	11.36 ± 2.08	HSC-T6 cells Wistar rats MCD diet-induced MASH mice	Intravenous	Li et al. (2019)
	CO	Micelle	Styrene maleic acid copolymer	109	Palmitic acid and Oleic acid-treated AML12 cells HF-MCD diet-induced MASH mice	Intravenous	Cui et al. (2023)
	Nifedipine	Nanoparticle	PLGA	210	Palmitic acid-treated HepG2 cells HFD-induced MASLD mice	Intravenous	Lee et al. (2019)
Anti-inflammatory drug	Baicalin	Nanoliposome	Soy lecithin and cholesterol	81.41	MCD diet-induced MASLD mice	Oral	Liu et al. (2020)
	Vitexin	Nanoliposome	DPPC and cholesterol	155	CCL4/Urethane co-induced liver cirrhosis rats	Intravenous and oral	Farooq et al. (2022)
	MCC950	Nanoliposome	PEG	151 ± 1	THP-1 cells	NA	Negro et al. (2023)
	short chain C6-Ceramide	Nanoliposome	PEG	90	human hepatic stellate cells (hHSCs) MCD diet-induced MASLD mice	Intravenous	Zanieri et al. (2020)
	Chrysin	Nanoliposome	Egg yolk lecithin and cholesterol	121 ± 8	MCD diet-induced MASH mice	Oral	Liu et al. (2023)
Nucleic acid drug	HMGB1-siRNA	Nanoliposome	DSPE-PEG-Man, DLinMC3-DMA, PEG-DMG, DSPC and cholesterol	100	RAW264.7 macrophages HFD-induced MASH mice	Intravenous	Zhou et al. (2022)
	CD98 siRNA	Nanoparticle	PLA	275	HepG2 cells HFD-induced MASLD mice	Intravenous	Canup et al. (2017)
	microRNA 146b	Nanoparticle	Lac-PDMAEMA	350	Palmitic acid-treated HepG2 and AML12 cells MCD diet-induced MASLD mice	Intravenous	He et al. (2018b)
	IL-22 gene	Nanoparticle	Chitosan, penetratin and DSPE-PEG2000	100	HepG2 and Huh7 cells HFD-induced MASLD mice	Intravenous	Zai et al. (2019)

abbreviations in the table: MASLD, non-alcoholic fatty liver disease; MASH, non-alcoholic steatohepatitis; HFD, high fat diet; FFA, free fatty acid; SNEDDS, self-emulsifying drug delivery systems; MCD, methionine choline deficiency; NA, not applicable. CCL4, carbon tetrachloride. CDAA, choline deficient amino acid; CO, carbon monoxide; HF-MCD, high fat and methionine choline deficiency; IL-22, interleukin-22; DPPC, dipalmitoylphosphatidylcholine; POPC, 1-palmitoyl-2-oleoyl lecithin. PLGA, poly (D, L-lactic-co-glycolic acid). PEG, poly (ethylene glycol); DOPC, 1,2-Dioleoyl-sn-glycero-3-phosphocholine; PBE, 4-aminophenylboronic acid pinacol ester; PFM, phenformin; PLA, polylactic acid. Others are fixed names or conventions.

### 2.1 Insulin sensitizer and lipid-lowering drug

**FIGURE 1:**
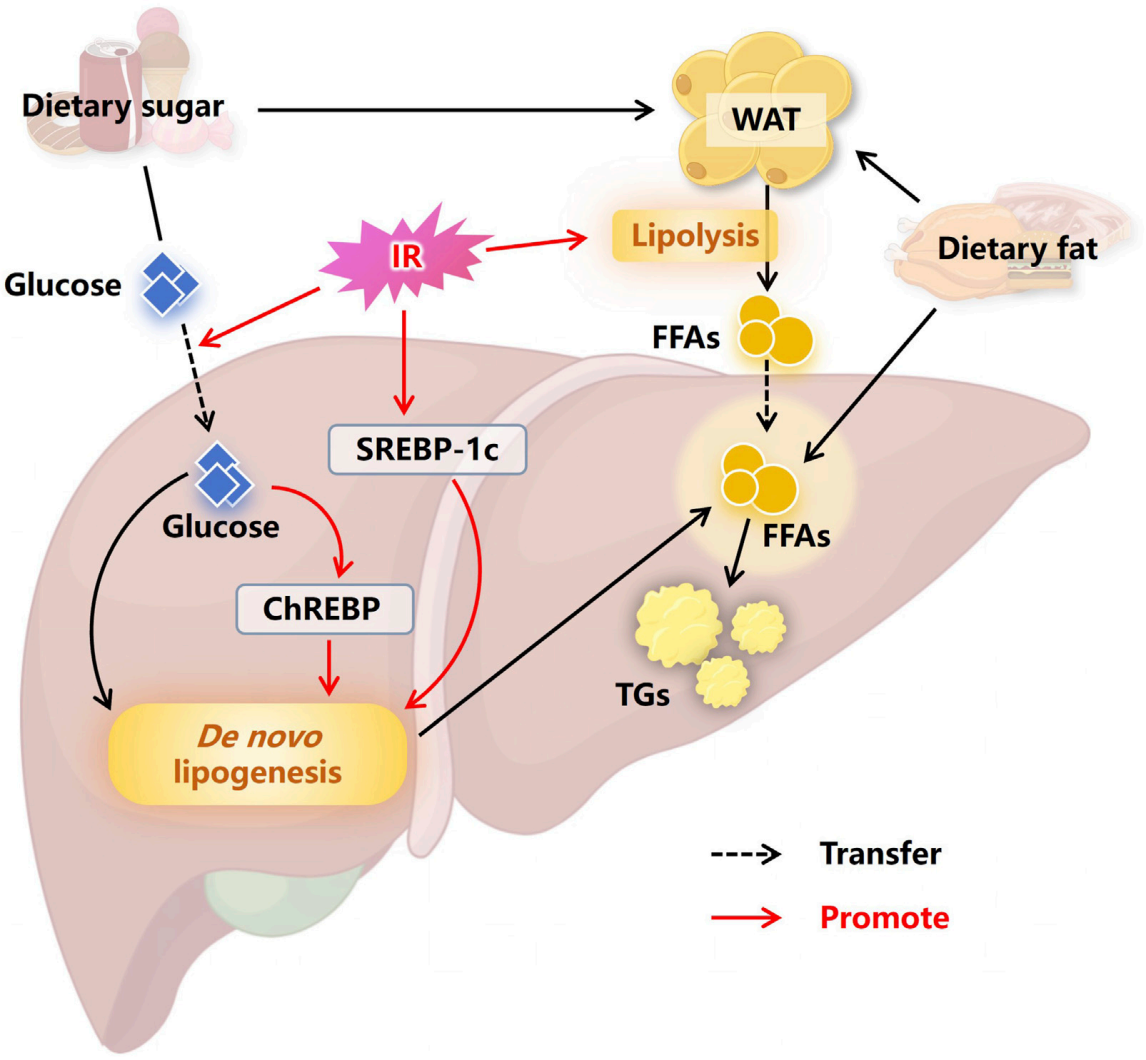
Insulin resistance promotes hepatic lipid accumulation. The excess energy obtained from a high-sugar and high-fat diet is usually stored in the form of fat in WAT. IR causes accelerated lipolysis in WAT, with large amounts of FFAs entering the liver, leading to ectopic fat storage. At the same time, IR promotes the entry of carbohydrates into the liver, where the carbohydrates are converted into fatty acids through DNL, further promoting hepatic lipid accumulation. In addition, SREBP-1c and ChREBP are activated by IR and the high intracellular concentrations of glucose, respectively, which advances DNL and ultimately advance the occurrence of MASLD. Abbreviations: WAT, white adipose tissue. IR, insulin resistance. FFAs, free fatty acids. SREBP-1c, sterol regulatory element binding protein 1c. ChREBP, carbohydrate responsive element binding protein. TGs, triglycerides. MASLD, non-alcoholic fatty liver disease.

When energy intake exceeds expenditure, the excess energy is stored as fat, primarily in lipid droplets within white adipose tissue (WAT) (Zechner et al., 2009; Byrne and Targher, 2015). MASLD is a typical disorder of ectopic fat storage. In the liver, more than half of the excessive accumulation of TGs comes from WAT, approximately one-third from *de novo* lipogenesis (DNL), and 15% from a high-fat and high-sugar diet (Machado and Cortez-Pinto, 2014; Heeren and Scheja, 2021). IR plays a crucial role in this process of ectopic fat deposition in the liver, as depicted in Figure 1. Insulin has an anti-lipolytic effect, promoting the esterification and storage of fatty acids (Tanase et al., 2020). IR diminishes the anti-lipolytic activity of insulin, leading to accelerated lipolysis in WAT. Large amounts of free fatty acids (FFAs) release into the liver, where they are subsequently stored ectopically as TGs (Haemmerle et al., 2006; Samuel and Shulman, 2012). Furthermore, IR has been proved to activate sterol regulatory element-binding protein 1c (SREBP-1c) (Dong et al., 2020), while the increase in intracellular glucose concentration caused by IR can activate carbohydrate response element-binding protein (ChREBP) (Denechaud et al., 2008; Lawitz et al., 2018), both of which are key proteins in regulating DNL (Ferré and Foufelle, 2010; Song et al., 2018). Therefore, IR significantly promotes intracellular DNL levels, facilitating further hepatic lipid accumulation (Lawitz et al., 2018; Luukkonen et al., 2022).

Improving insulin resistance and reducing lipid accumulation have become important breakthroughs for researchers in developing drugs for MASLD. For example, Silibinin, the main active component of *Silybum marianum*, has been widely used in the clinical treatment of liver diseases and has shown potential in treating MASLD (Federico et al., 2017; Liu et al., 2019). However, the therapeutic potential of Silibinin is limited by its poor water solubility and low bioavailability (Bi et al., 2019). A study constructed lipid nanoparticles loaded with Silibinin (Sil-Lip), and then evaluated the therapeutic effect of Sil-Lip by using HFD-induced MASLD mice and FFA-stimulated HepG2 cells (Yan et al., 2023). The results indicated that Sil-Lip effectively alleviated insulin resistance and lipid metabolism disorders, with good gastrointestinal stability, mucosal penetration, oral absorption, and bioavailability (Yan et al., 2023).

**FIGURE 2:**
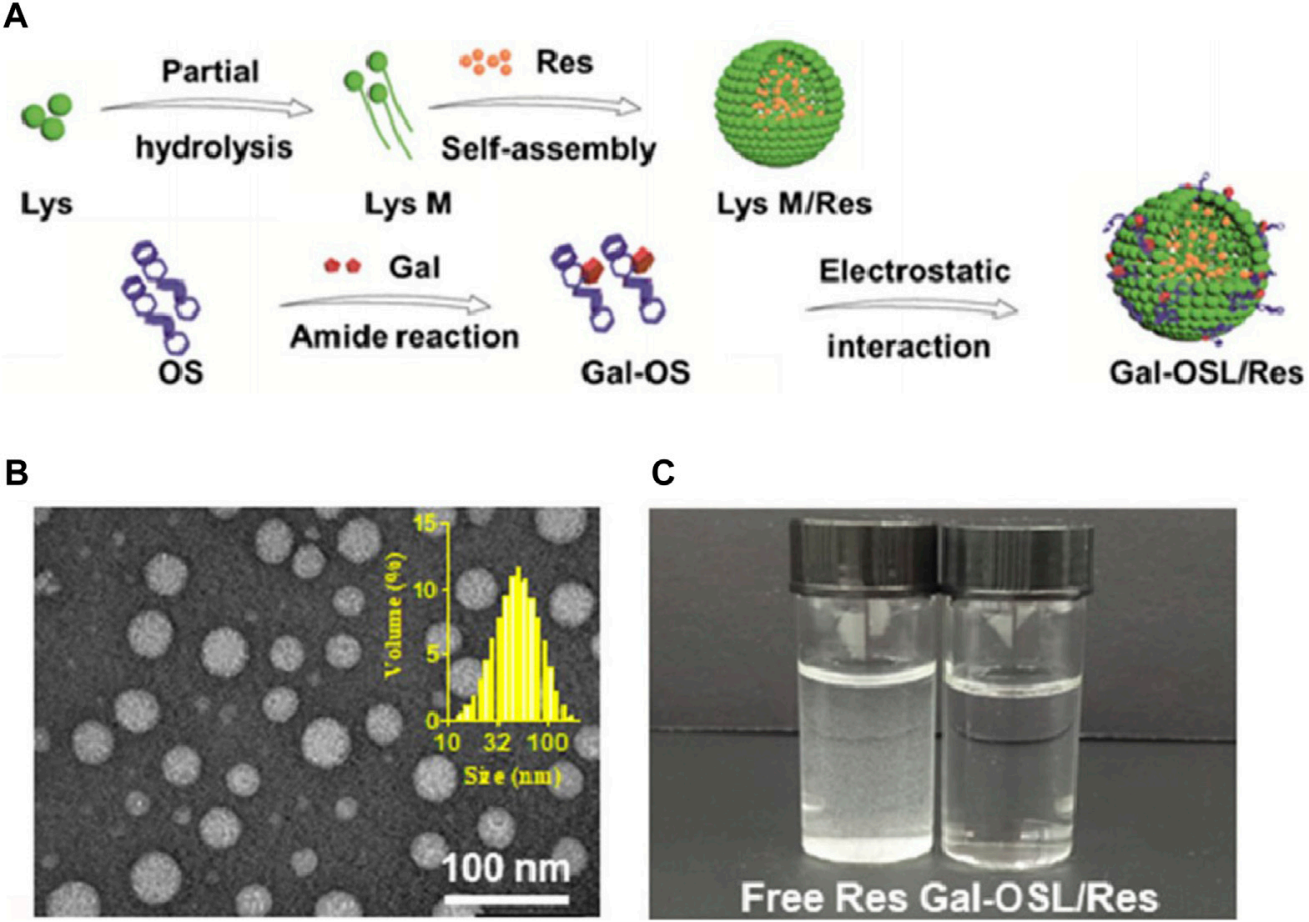
Characterization of Gal-OSL/Res nanocarriers (Teng et al., 2019). **(A)** Schematic representation of Gal-OSL/Res. **(B)** Transmission electron microscope (TEM) images and dynamic light scattering (DLS) size distribution of Gal-OSL/Res in aqueous solution. **(C)** Free Res and Gal-OSL/Res in aqueous solution with equal Res concentration of 0.1 mg/mL.

Resveratrol is another natural product with potential therapeutic effects on MASLD, mainly found in red grapes and nuts (Tennen et al., 2012). It has been found to alleviate hepatic insulin resistance and metabolic disorders (Faghihzadeh et al., 2015). However, resveratrol also suffers from poor water solubility and susceptibility to degradation in the intestine (Gescher and Steward, 2003). Moreover, after intravenous injection, resveratrol is almost undetectable in the liver (Liang et al., 2013), indicating that increasing its effective accumulation in the liver is a prerequisite for its treatment of MASLD (Teng et al., 2019). A team developed resveratrol nanocarriers (Gal-OSL/Res) targeted to the liver using oxidized starch lysozyme (OSL) as a nanocarrier and covalently linking it with galactose (Gal), which successfully increased the liver uptake of resveratrol (Teng et al., 2019). The preparation process and characterization of Gal-OSL/Res are shown in Figure 2. Cell and animal experiments of this study demonstrated that Gal-OSL/Res effectively improved lipid deposition and insulin resistance by regulating the AMPK/SIRT1/FAS/SREBP1c signaling pathway, thereby reliably reversing MASLD (Teng et al., 2019).

The mechanistic target of rapamycin complex 1 (mTORC1) has been found to be a key checkpoint in the pathogenesis of SREBP-1c mediated DNL and MASLD (Lee et al., 2017; Kim and Guan, 2019). As an inhibitor of mTORC1, rapamycin (RAPA) can alleviate lipid metabolism disorders, significantly improving insulin resistance and hepatic steatosis (Zhou and Ye, 2018). However, its severe adverse reactions (Bee et al., 2018) and lower bioavailability (Othman et al., 2016) restrict its clinical use. Zhao et al. (2020) designed and developed polymer nanoparticles to encapsulate RAPA named NP-RAPA, with monomethoxy-poly (ethylene glycol) (mPEG) as the hydrophilic shell and poly (D, L-lactic-co-glycolic acid) (PLGA) as the backbone structure. Compared to free RAPA, NP-RAPA significantly improved the lipid homeostasis by reducing SREBP-1c mediated DNL, thus decreased the accumulation of low-density lipoproteins in the liver of diet-induced MASLD mice (Zhao et al., 2020).

*Schisandra chinensis* (SC) is a traditional IR Chinese medicinal herb commonly used to treat liver damage and liver dysfunction (Panossian and Wikman, 2008). Previous studies have reported that SC could inhibit cell differentiation and lipid accumulation in 3T3-L1 preadipocytes (Park et al., 2012), but its clinical applicability is limited by poor water solubility. Deoxyschizandrin (DS) is the main active ingredients of SC. Liu et al. (2018) prepared lipid nanoparticles loaded with DS (DS-lipo), with a diameter of approximately 73 nm. This formulation exhibited remarkable inhibition of lipid droplet accumulation without affecting cell growth, providing a promising strategy for the treatment of MASLD and lipid-related diseases (Liu et al., 2018).

### 2.2 Antioxidant

**FIGURE 3:**
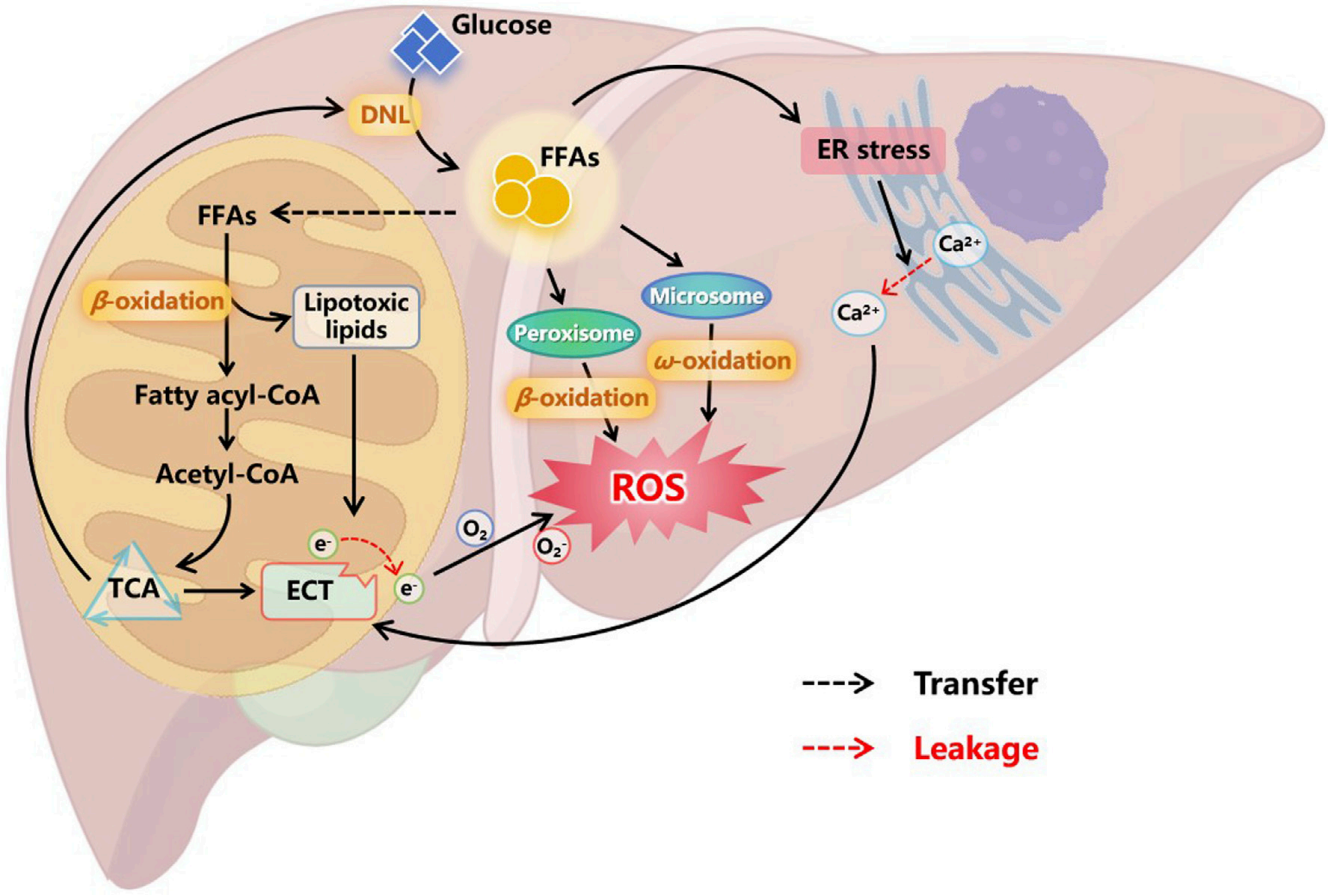
Oxidative stress in the progression of MASLD. Excessive FFAs in liver cells enhance compensatory FAO. In addition to oxidative degradation through peroxisomes and microsomes, more FFAs enter mitochondria and convert to acetyl-CoA to access the TCA cycle. However, overloaded FAO leads to the accumulation of lipotoxic lipids, which can damage the ETC on the mitochondrial inner membrane, cause electron leakage, and result in the production of a large amount of ROS. Moreover, FFAs can also cause ER stress, leading to the leakage of calcium ions stored in the ER into the cytoplasm. These free ions further damage the integrity of ETC, causing sustained ROS production. Oxidative stress in cells affects multiple normal physiological functions, ultimately forming a vicious cycle that prompts liver cell damage and inflammation. Abbreviations: DNL, *de novo* lipogenesis. FFAs, free fatty acids. ER, endoplasmic reticulum. TCA, tricarboxylic acid cycle. ETC, electronic transfer chain. ROS, reactive oxygen species.

Under physiological conditions, excess carbohydrates are converted into fatty acids via DNL and further stored in cells in the form of TGs. When needed, TGs provide energy to the body through fatty acid oxidation (FAO) (Ameer et al., 2014). The FAO system comprises mitochondria and peroxisomes (primarily β-oxidation), as well as microbodies (mainly ω-oxidation) (Pyper et al., 2010). Excessive accumulation of FFAs in the liver caused by various factors will enhance the compensatory ability of cellular FAO, leading to the increase of the production of FAO-originated reactive oxygen species (ROS) (Chen Z. et al., 2020a). Meanwhile, overloaded FAO can cause accumulation of lipotoxic intermediates, resulting in mitochondrial damage, particularly impairing the normal function of the electron transport chain (ETC) (Gao et al., 2004; Masarone et al., 2018). The imbalance between the compensatory enhancement of FAO and the damaged ETC in mitochondria lead to electron leakage, which contributes to a large amount of ROS production (Rolo et al., 2012; Sunny et al., 2017), as illustrated in Figure 3. Additionally, the amphipathic nature of FFAs facilitates their incorporation into the inner membrane of mitochondria, increasing membrane fluidity, which may further promote electron leakage (Stillwell et al., 1997; Chen Z. et al., 2020a). Disruption of the oxidative-reductive homeostasis within liver cells further affects the normal process of FAO and exacerbates mitochondrial damage, forming a vicious cycle, resulting in sustained elevation of ROS levels, worsening liver injury, and promoting disease progression to more severe stages.

**FIGURE 4:**
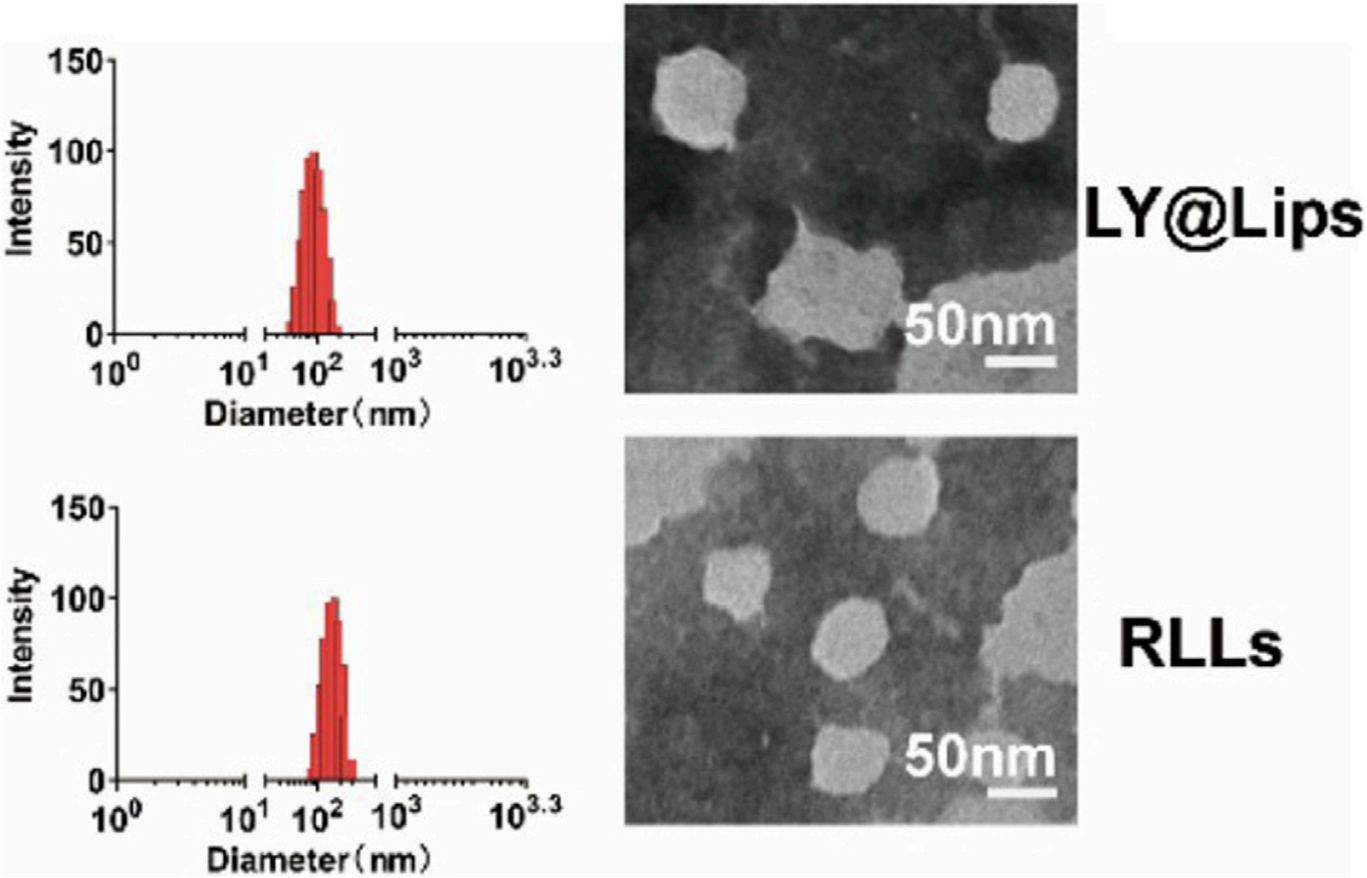
The intensity-based size distribution by DLS analysis and morphological characteristics captured by TEM of LY@Lips (LY294002 encapsulated liposomes) and RLLs (Xin et al., 2023).

The consumption of intracellular ROS through medication can partially alleviate oxidative stress and control the progression of MASLD. However, the pathology of MASLD is more likely due to the dysregulation of redox signaling pathways rather than simply an increase in ROS concentration (Chen Z. et al., 2020a). Therefore, drugs targeting specific ROS sources or specific cellular redox events in the liver may be more effective in treating MASLD. Xin et al. (2023) designed a kind of nanoliposomes targeting hepatic stellate cells (HSCs) for treating MASLD. In brief, this study first utilized a sulfone linker (HA-TK-ORD) with ROS scavenging capability to combine oridonin with hyaluronic acid (HA). Then, HA-TK-ORD was coated on the surface of liposomes encapsulating LY294002, forming liposomes with a diameter of approximately 125 nm, named RLLs (Xin et al., 2023). CD44, highly expressed in HSCs (Li et al., 2020), is a well-known receptor for HA (Bhattacharya et al., 2017), and LY294002 is a widely used PI3K inhibitor (Garlich et al., 2008), while oridonin is an active ingredient isolated from *Rabdosia rubescens* (Cummins et al., 2018), which can inhibit the AKT/mTOR/NF-κB pathway (He H. et al., 2018a). Therefore, RLLs are CD44-mediated, ROS-responsive liposomes targeting HSCs. The intensity-based size distribution and morphological characteristics of RLLs is shown in Figure 4. After intravenous injection of RLLs into MASLD mice, due to the ROS reactivity of HA-TK-ORD and the targeting property of HA, the liposomes released LY294002 and oridonin in HSCs, subsequently regulating metabolic dysfunction in MASLD through multiple perturbations acting on the PI3K-AKT-mTOR-NF-κB axis (Xin et al., 2023).

**FIGURE 5:**
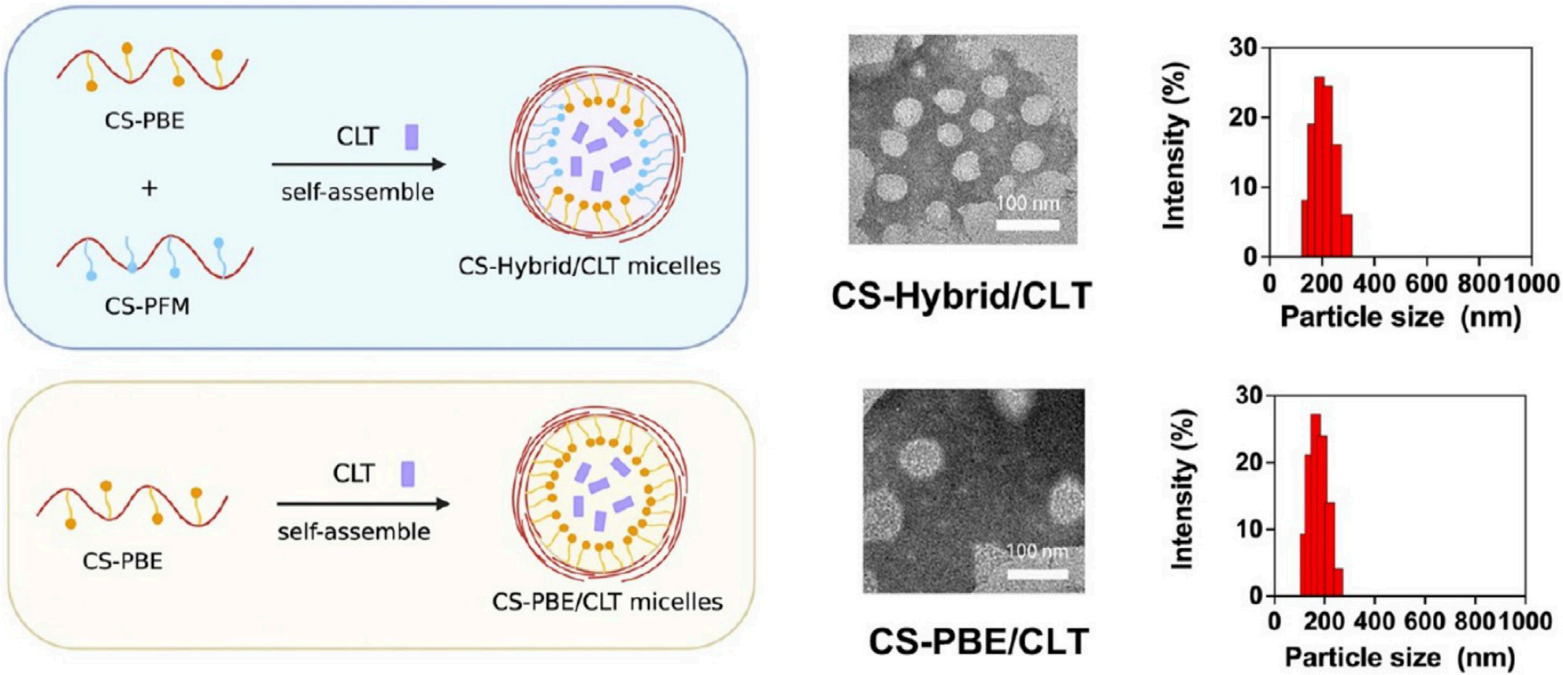
Representative TEM images and size distributions of CS-Hybrid/CLT and CS-PBE/CLT (Pan et al., 2023).

Celastrol (CLT), a pentacyclic triterpenoid extracted from *Tripterygium wilfordii Hook. f.* (Choi et al., 2016), is believed to possess broad pharmacological activities such as anticancer, antioxidant, and anti-inflammatory effects (Wang et al., 2020). Studies have also found that CTL may induce weight loss through various mechanisms, including sensitization to leptin and appetite control (Greenhill, 2015; Liu et al., 2015), suggesting CTL as a potential anti-MASLD drug. A research team synthesized two derived amphoteric materials using chondroitin sulfate (CS), which has a strong affinity for CD44 (Khan et al., 2020), including CS coupled with 4-aminophenylboronic acid pinacol ester (CS-PBE) and CS coupled with phenformin (CS-PFM), as shown in Figure 5. CTL was then encapsulated in mixed micelles self-assembled from CS-PBE and CS-PFM, resulting in CS-Hybrid/CLT nanoparticles with the ability to target the liver (Patouraux et al., 2017), scavenge ROS (Pan et al., 2023), and enhance the uptake efficiency of micelles by fat cells (Sogame et al., 2009). In the HFD-induced MASLD rat model, CS-Hybrid/CLT micelles significantly reduced hepatic lipid accumulation and levels of FFAs, markedly improved oxidative stress, and downregulated hepatic inflammation (Pan et al., 2023).

**FIGURE 6:**
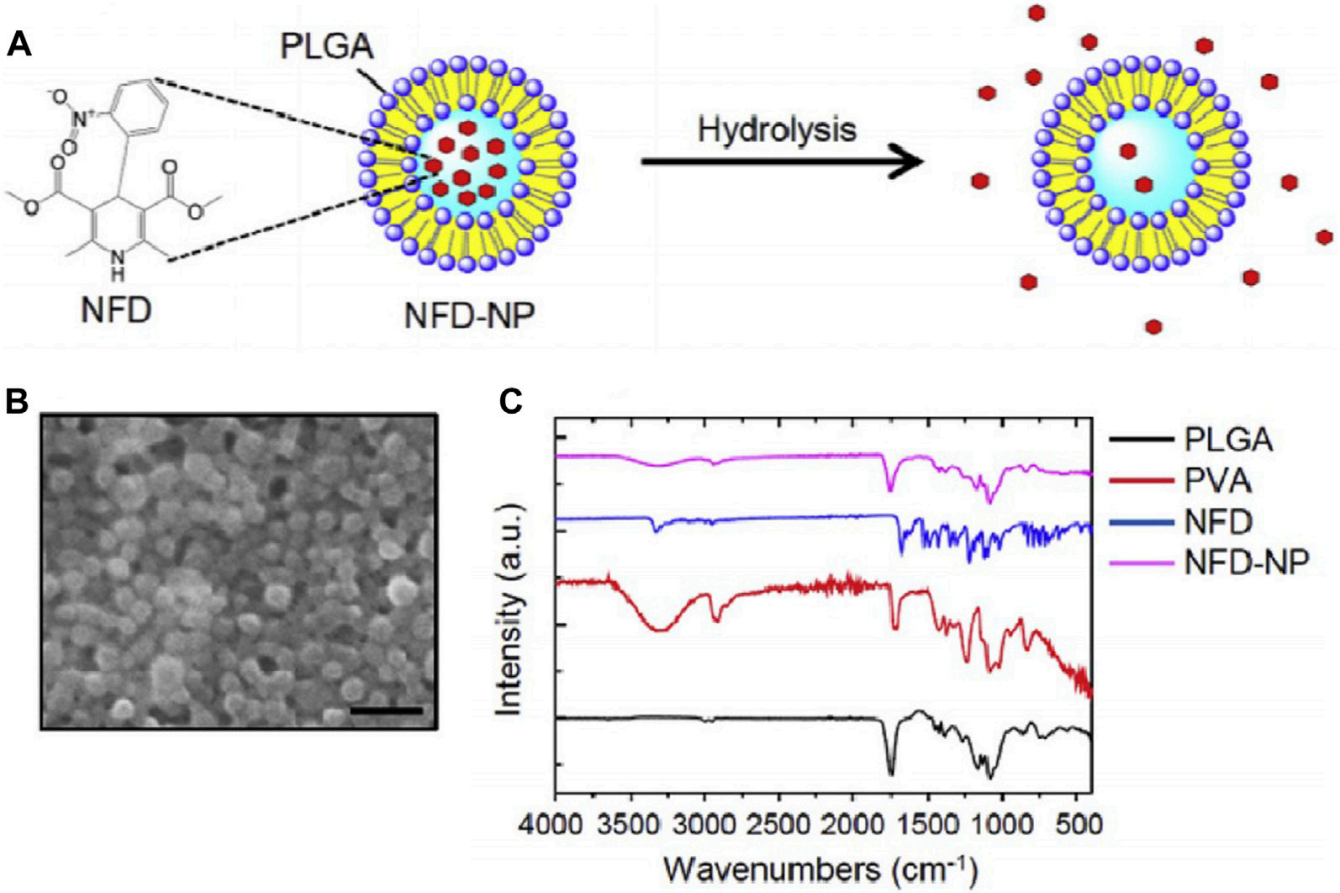
Characterization of NFD-NPs (Lee et al., 2019). **(A)** NPs encapsulating NFD are prepared with PLGA and poly (vinyl alcohol) (PVA) as a surfactant. **(B)** The morphology of NFD-NPs was characterized by SEM. Scale bar, 500 nm. **(C)** Fourier transform-infrared spectroscopy spectra of PLGA, PVA, NFD, and NFD-NPs.

In addition to causing mitochondrial damage, excessive FFAs in the liver also act on the endoplasmic reticulum (ER), leading to ER stress (Figure 3). Subsequently, accumulation of misfolded and unfolded proteins in the ER lumen activates the unfolded protein response to restore ER homeostasis (Malhi and Kaufman, 2011). Moreover, the ER is a major storage site for intracellular calcium (Sugimoto et al., 2010), and ER stress can cause calcium leakage into the cytoplasm. These free calcium ions may act on the mitochondrial membrane, affecting mitochondrial ETC and inducing further metabolic disturbances and cell death (Griffiths and Rutter, 2009; Yin et al., 2015). Nifedipine (NFD) is a calcium channel blocker approved by the FDA for controlling hypertension, angina, and arrhythmias (Murdoch and Brogden, 1991). Lee et al.(2019) prepared nanoparticles loaded with NFD (NFD-NPs), as shown in Figure 6, to reduce protein aggregates and ER stress induced by palmitate. NFD-NP had illustrated no cytotoxicity in HepG2 cells. Additionally, the nanoparticles increased the oral bioavailability of NFD and prolonged its release *in vivo*. After intravenous injection of NFD-NPs into HFD-induced MASLD mice, the nanoparticles effectively suppressed lipid metabolism disorders (Lee et al., 2019).

### 2.3 Anti-inflammatory drug

When the accumulation of lipids in hepatocytes exceeds the compensatory capacity of mitochondrial FAO, it will bring about lipid toxicity and the extensive generation of ROS, subsequently causing liver damage (Fromenty and Roden, 2023). Then, damaged hepatocytes release damage-associated molecular patterns (DAMPs), which, together with pathogen-associated molecular patterns (PAMPs) released by gut microbiota, activate immune cells in the liver, such as Kupffer cells (KCs), through pattern recognition receptors (PRRs) (Takeuchi and Akira, 2010; Kubes and Mehal, 2012). The activated KCs regulate the inflammatory response in the liver microenvironment by secreting pro-inflammatory cytokines, promoting the further progression of the disease to MASH status (Guo et al., 2022). Toll-like receptor 4 (TLR4) is a critical member of PRRs, playing a key role in the innate immune response, and has been found to be significantly upregulated on the surface of KCs in MASH mice (Kesar and Odin, 2014; Sharifnia et al., 2015). Many studies consider the inhibition of the TLR4 signaling pathway as a potential therapeutic target for MASH.

A flavonoid known as chrysin (CH) can be extracted from passion fruit, propolis, and honey (Pisonero-Vaquero et al., 2015; Van De Wier et al., 2017). This natural product has been found to possess multiple biological properties, such as liver protection, immune regulation, and antioxidant effects (Mani and Natesan, 2018; Naz et al., 2019), and has been proven to improve HFD-induced hepatic steatosis (Feng et al., 2014). Feng et al. (2014) discovered that CH could ameliorate hepatic steatosis by modulating the status of macrophage M1/M2. However, the oral bioavailability of CH is only 0.003%–0.02% (Walle et al., 2001), severely limiting its clinical efficacy. Liu et al.(2023) improved the serum and liver concentration of CH significantly and ameliorated lipid accumulation in mice with MASH induced by a methionine-choline deficient (MCD) diet, through the preparation of CH nanoliposomes (CH-NL). Additionally, the study also found that CH-NL downregulated the activation of the TLR4 signaling pathway in the liver, significantly inhibiting the production of inflammatory cytokines and the infiltration of inflammatory cells in the liver of MASH mice (Liu et al., 2023). Another study utilized nanoliposomes to encapsulate baicalin, a natural flavonoid compound extracted from the root of *Scutellaria baicalensis Georgi*. They found that the nanoliposomes effectively increased the bioavailability of baicalin and reduced plasma transaminase, hepatocyte apoptosis, liver lipid accumulation, liver fibrosis, and the infiltration of neutrophils and macrophages in MASLD mice, with this effect also mediated by the inhibition of the TLR4 signaling cascade (Liu et al., 2020).

**FIGURE 7:**
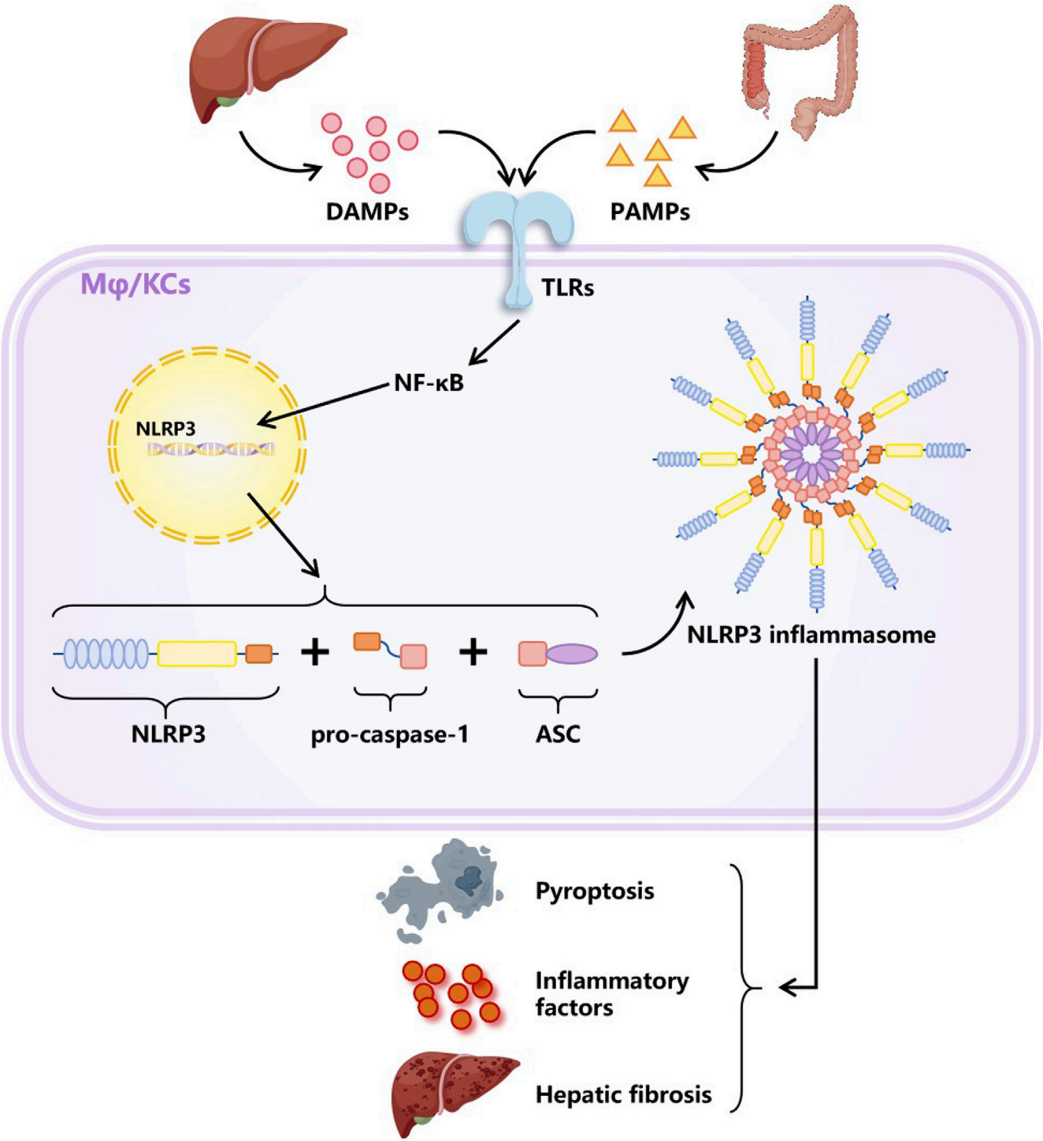
Classic activation way of NLRP3 inflammasome. DAMPs secreted by damaged liver cells together with PAMPs produced by gut microbiota activate intracellular signaling pathways by stimulating TLRs, resulting in the upregulation of NLRP3 expression. Afterwards, NLRP3 molecules recruited pro-caspase-1 and adapter protein ASC to assemble into NLRP3 inflammasomes. The activation of inflammasomes plays an important role in the progression of MASLD, as it can cause pyroptosis, exacerbated inflammation, and liver fibrosis. Abbreviations: DAMPs, damage related molecular patterns. PAMPs, pathogen activated molecular patterns. TLRs, toll like receptors. Mφ, macrophages. KCs, Kupffer cells (special macrophages in the liver). NLRP3, NLR family pyridin domain containing 3. ASC, a caspase admission domain.

Furthermore, the activation of inflammasomes is also of vital importance in the progression of MASH and has attracted wide attention in recent years. Inflammasome is a multi-protein complex, which is an emerging mediator of the interactions between host and inflammatory cells based on the activation of the NLR family pyrin domain containing 3 (NLRP3) (Mridha et al., 2017). When toll-like receptors (TLRs) recognize harmful signals such as PAMPs and DAMPs, NLRP3 expression will be upregulated, and adapter protein ASC and pro-caspase-1 will be recruited to assemble into inflammasomes, which is the classic activation mode of NLRP3 inflammasomes. The activation of NLRP3 inflammasomes ultimately induces pyroptosis, exacerbation of inflammation, and fibrosis (Schroder and Tschopp, 2010; Hara et al., 2013; Masters, 2013; Szabo and Petrasek, 2015), as shown in Figure 7. MCC950 is a small molecule inhibitor of the NLRP3 inflammasome, proven to have significant therapeutic effects in many NLRP3-driven inflammatory diseases (Scavo et al., 2020). However, MCC950 has a short plasma half-life and lacks targeting ability (Li et al., 2022). Frizzled protein 1 (FZD1), involved in the WNT signaling pathway, was found to be overexpressed on inflammasome-activated macrophages (Neumann et al., 2010). A study encapsulated MCC950 in PEG liposomes containing FZD1 specific antibodies to selectively target macrophages. The nanoparticles exhibited the characteristics of reducing inflammasome activation and inhibiting fibrosis formation in both *in vivo* and *in vitro* models, providing a potential therapeutic strategy for reversing inflammation and fibrosis in MASH (Negro et al., 2023).

### 2.4 Nucleic acid drug

In recent years, an increasing number of approved nucleic acid therapies have demonstrated extraordinary potential for treating diseases by targeting *in vivo* genes (Kulkarni et al., 2021). Widely used nucleic acid-based drugs include DNA drugs, nucleic acid aptamers, antisense oligonucleotides, messenger RNA, microRNA, small interfering RNA, and small activating RNA (Wang et al., 2023). The direct, effective, and long-lasting therapeutic effects make nucleic acid drugs a powerful weapon for treating various diseases, and their broad prospects in the treatment of MASLD are also being evaluated. However, the clinical application of these drugs still faces several challenges, including low efficiency of biofilm passage limited by the molecular weight and negative charge of nucleic acids, susceptibility to enzymatic hydrolysis or recognition and clearance by the immune system, and restriction of function by endocytosis after entering cells (Hou et al., 2021; Thangamani et al., 2021; Wang et al., 2024). Currently, modifying nucleic acid molecules to enhance stability, avoid immune system attack, and using nanomaterials as drug delivery carriers are common optimization methods (Hou et al., 2021; Zhou et al., 2021).

**FIGURE 8:**
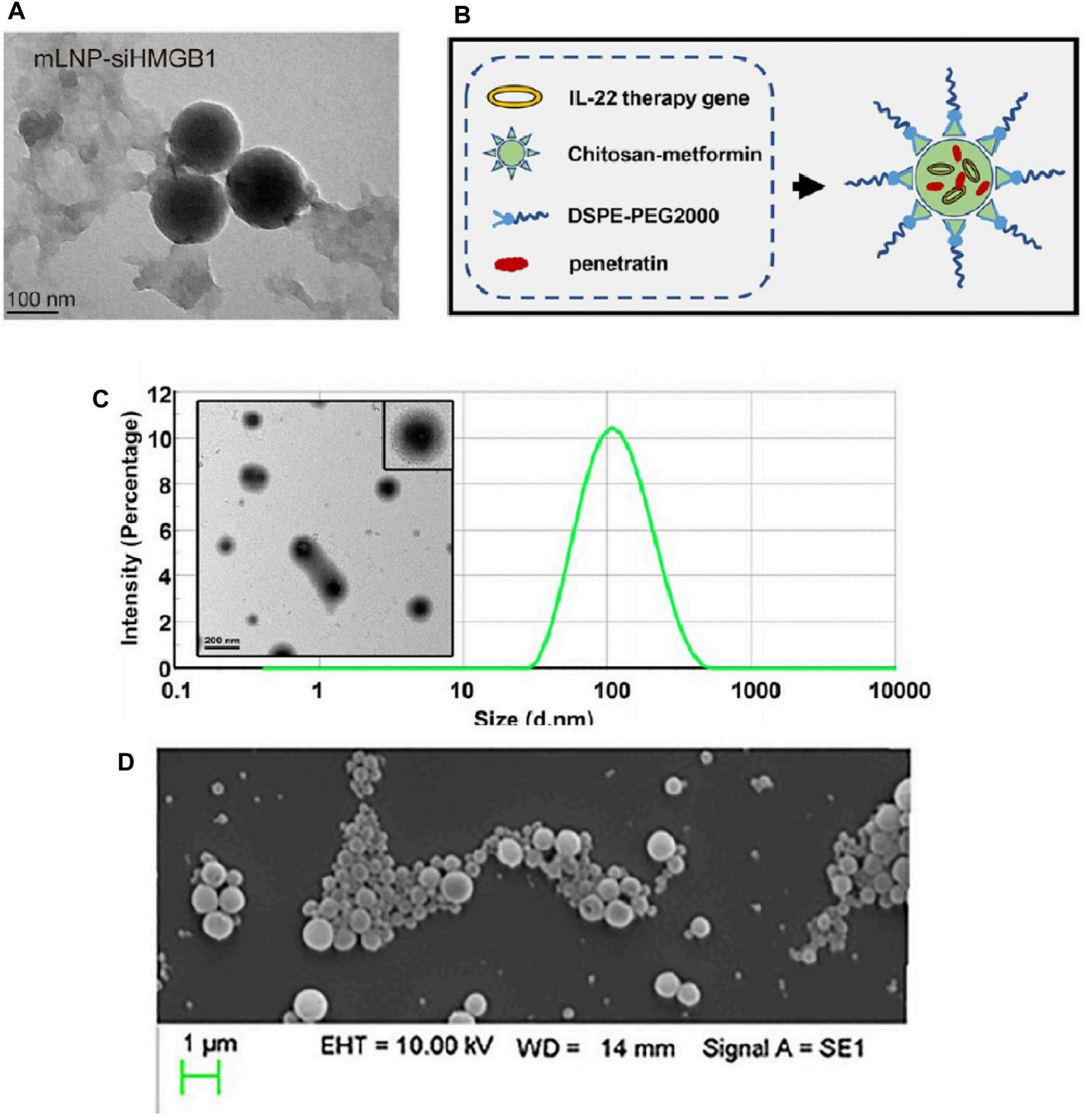
Characterization of some nanoparticles loaded with nucleic acid drugs. **(A)** The TEM image of mLNP-siHMGB1 (Zhou et al., 2022). Construction **(B)** and characterization **(C)** of CDPIA nanocomplexes (Zai et al., 2019). **(D)** SEM image of 500 μg/mL CD98 siRNA-loaded NPs suspension (Canup et al., 2017).

High mobility group protein B1 (HMGB1) is a highly conserved nuclear protein secreted by various cells, such as damaged liver cells, macrophages, monocytes, and dendritic cells (DCs), which belongs to the DAMPs family. Its key role in the progression of inflammation in MASH has led to widespread attention (Ge et al., 2018). HMGB1 and other inflammatory factors (e.g., TNF-α, IL-6) and chemokines (e.g., CCL2, CCL5) can promote the conversion of KCs to pro-inflammatory M1 type (Krenkel et al., 2018; Schuster et al., 2018) and recruit a large number of activated bone marrow-derived macrophages (BMMs) into the liver, releasing more HMGB1 and chemokines, forming a vicious cycle (Tacke, 2017; van der Heide et al., 2019). Many studies have shown that blocking excess HMGB1 can reduce inflammatory liver diseases (Li et al., 2018; Zhang et al., 2020). Zhou et al. (Zhou et al., 2022) constructed a mannose-modified HMGB1-siRNA loaded stable nucleic acid lipid particle delivery system (mLNP-siHMGB1), as shown in Figure 8A. Thanks to mannose receptor-mediated targeting of liver macrophages, mLNP-siHMGB1 successfully silenced HMGB1 protein expression, and regulated liver macrophages towards anti-inflammatory M2 phenotype differentiation, which effectively reduced lobular inflammation and large vesicular fatty degeneration of the liver, restoring liver function in MASH mice to normal levels (Zhou et al., 2022).

Interleukin-22 (IL-22) is a novel cytokine of the IL-10 family, which has shown evidence in recent studies to alleviate metabolic syndrome in obese mice, improve insulin resistance, and regulate the expression of genes related to lipogenesis (Hasnain et al., 2014; Wang et al., 2014). However, the improvement of MASLD symptoms with safe doses of IL-22 is limited, while higher concentrations of IL-22 may produce off-target toxicity (Pan et al., 2013) or induce cachexia, such as thymic atrophy and proximal tubule lesions (Liang et al., 2010; Park et al., 2015). Therefore, optimizing the systemic distribution and circulation time of IL-22 through structural or formulation modifications is of importance to enhance its efficacy against MASLD and reduce side effects (Zai et al., 2019). A study first coupled metformin with chitosan to develop a novel poly-metformin carrier (CM), which not only has advanced gene delivery efficiency (Luo et al., 2016; Zhao et al., 2016) but also the intrinsic therapeutic effect of metformin on MASLD. Subsequently, through electrostatic interactions, CM accompanied by transfection protein and DSPE-PEG2000 self-assembled with the IL-22 gene to form a stable nanocomplex, named CDPIA (138). Among the complex, the transfection protein is a cell-penetrating auxiliary agent with excellent permeability and delivery capacity (Christiaens et al., 2005; Liu et al., 2016), and DSPE-PEG2000 is an auxiliary lipid that can reduce non-specific interactions and promote endosomal release of the plasmid (Chen W. et al., 2018a). The construction and characterization of CDPIA nanocomplexes have been shown in Figures 8B, C. The results of the study indicated that CDPIA significantly alleviated hepatic steatosis in the HFD-induced MASLD mouse model, and long-term injection of CDPIA did not show systemic toxicity in mice, suggesting that CDPIA could effectively control the local secretion of IL-22 and reduce its off-target toxicity (Zai et al., 2019).

CD98 is a pro-inflammatory receptor involved in many inflammation-related diseases and various cancers (Kucharzik et al., 2005; Kaira et al., 2009; Nguyen et al., 2011; Xue et al., 2012), so some research teams believed that blocking the expression of CD98 in the liver in the early stages of inflammation may control the progression of MASLD to cirrhosis or HCC(148). The team used a double emulsion/solvent evaporation technique (Laroui et al., 2011; Laroui et al., 2014) to encapsulate CD98 siRNA in poly-lactic acid (PLA) nanoparticles, ensuring the release of siRNA in the cytoplasm by pre-electrostatically binding CD98 siRNA with short-chain polyethyleneimine (PEI) (Canup et al., 2017), as shown in Figure 8D. The results showed that the nanoparticles loaded with CD98 siRNA dramatically reduced all markers of MASLD induced by HFD in mice, including levels of alanine transaminase in the blood, lipid accumulation, evidence of fibrosis, and pro-inflammatory cytokines (Canup et al., 2017).

## 3 Summary and outlook

**FIGURE 9:**
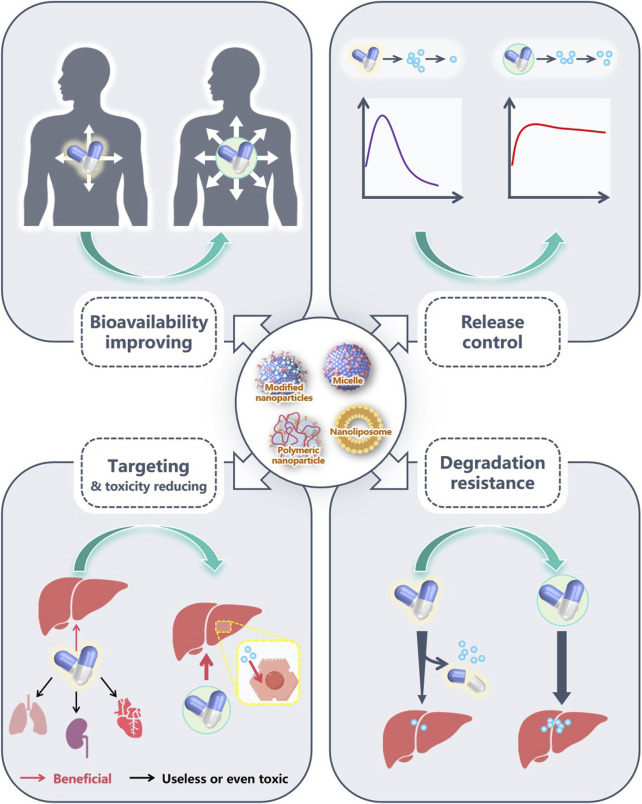
The advantages of NDDS in MASLD pharmacotherapy. Nanomaterials, through their universal amphiphilic structure, effectively enhance the biocompatibility of poorly water-soluble drugs, increase the concentration of these drugs *in vivo*, and help them fully exert their pharmacological effects. Some nanomaterials have good sustained-release or sustained release capabilities, which can also fully exert the effects of drugs. Several drugs have poor targeting ability, and some even have certain toxicity to other organs. By adding modified molecules, many nanomaterials exhibit excellent targeting effects, accurately delivering drugs to target organs or cells. In addition, as drugs are easily degraded during transportation, good encapsulation of nanomaterials can reduce drug loss during transportation and fully exert their efficacy.

Limited by the incomplete elucidation of the pathogenesis of MASLD, various drugs targeting the causative factors of MASLD are still mainly relied upon to control the disease progression. Therefore, how to fully utilize the efficacy of these drugs has become a hot topic in current research. Although most trials are still in the animal testing phase, it is undoubtedly an exciting endeavor to use nanomaterials to enhance the therapeutic effects of MASLD drugs or to reduce their toxicity. Whether liposomes or micelles, drug-loaded nanoparticles effectively increase the solubility of drugs, allowing their biological activity to be fully exhibited. Besides, by attaching specific targeting molecules (e.g., Gal, HA, CS) to the nanoparticles, drugs are released and act at specific sites, not only enhancing therapeutic effects but also downgrading toxic side effects on other tissues. In recent years, as more pathogenic mechanisms have been discovered, some gene-targeting drugs have gradually been developed. These drugs are usually prone to degradation and off-target effects, making biocompatible nanomaterials an appropriate choice for protecting and transporting them. The optimization role of NDDS in the treatment of MASLD drugs is illustrated in Figure 9.

Research on MASLD drug nanocarriers has shown remarkable prospects, but there is still a need for more in-depth and comprehensive exploration before clinical application. Moreover, most studies have only focused on the pharmacokinetics of the encapsulated drugs, while more research is needed on the toxicokinetics and long-term safety of the drug carriers themselves on the blood and different organs (Najahi-Missaoui et al., 2020). Additionally, the stability of some materials needs further optimization. For example, micelle systems sometimes disintegrate in biological fluids, leading to premature drug release (Moghtadaie et al., 2023). Meanwhile, polymer nanoparticles, due to their generally larger size, still face challenges in cellular uptake and tissue penetration. The complicated production process of polymer nanoparticles also limits their large-scale industrial production to some extent (Hua et al., 2018), which is disadvantageous for their clinical applications.

In summary, the research combining NDDS with the treatment of MASLD is still in its initial stages, and while exciting interim results have been achieved, more in-depth understanding and long-term planning by researchers are required.

## Figures and Tables

**Table:** 

**NDDS**	Nano-Drug Delivery Systems
**MASLD**	Metabolic Dysfunction-Associated Steatotic Liver Disease
**MASH**	Metabolic Dysfunction-Associated Steatohepatitis
**HCC**	Hepatocellular Carcinoma
**TGs**	Triglycerides
**IR**	Insulin Resistance
**HFD**	High-Fat Diets
**WAT**	White Adipose Tissue
**DNL**	*de novo* Lipogenesis
**FFAs**	Free Fatty Acids
**SREBP-1c**	Sterol Regulatory Element-Binding Protein 1c
**ChREBP**	Carbohydrate Response Element-Binding Protein
**OSL**	Oxidized Starch Lysozyme
**Gal**	Galactose
**mTORC1**	The Mechanistic Target Of Rapamycin Complex 1
**mPEG**	Monomethoxy-Poly (ethylene glycol)
**PLGA**	Poly (D, L-lactic-co-glycolic acid)
**FAO**	Fatty Acid Oxidation
**ROS**	Reactive Oxygen Species
**ETC**	Electron Transport Chain
**HSCs**	Hepatic Stellate Cells
**HA**	Hyaluronic Acid
**CS**	Chondroitin Sulfate
**ER**	Endoplasmic Reticulum
**DAMPs**	Damage-Associated Molecular Patterns
**PAMPs**	Pathogen-Associated Molecular Patterns
**KCs**	Kupffer Cells
**PRRs**	Pattern Recognition Receptors
**TLR4**	Toll-Like Receptor 4
**MCD**	Methionine-Choline Deficient
**NLRP3**	NLR Family Pyrin Domain Containing 3
**TLRs**	Toll-Like Receptors
**FZD1**	Frizzled Protein 1
**HMGB1**	High Mobility Group Protein B1
**DCs**	Dendritic Cells
**BMMs**	Bone Marrow-Derived Macrophages
**IL-22**	Interleukin-22
**PEI**	Polyethyleneimine
